# 

**Published:** 1869-08

**Authors:** 


### CONTENTS.

ORIGINAL COMMUNICATIONS.

Ethics ... 317
Microscopy of the Dentinal Tubuli ... 324
How to Conduct a Dental Practice ... 331
Address of Dr. W. W. Allport, before the Illinois State Microscopical Society ... 341

PROCEEDINGS OF SOCIETIES.

Report of Discussions of the Mississippi Valley Dental Association, held March 3, 1869 ... 345
Hudson River Association of Dental Surgeons ... 348
Correction ... 349

SELECTIONS.

Fatal Case of Tetanus ... 350
Bromide of Potassium for the Troubles of Teething ... 351
Insanity Cured by the Removal of Carious Teeth ... 353
An Improved Syringe-Pipe for Hypodermic Injection ... 355
Influence of Tobacco upon the Circulatory System ... 356
Amaurosis Caused by Crowding of Teeth ... 356
Spectrum Lines ... 357
The Nerves of the Heart ... 357
Food in the Reign of Henry the Seventh ... 358
Purification of Tannin ... 358

EDITORIAL.

Tooth Powders, Etc ... 359
Resignation—Appointment ... 359
Sensitive Dentine in a Pulpless Tooth ... 360
Correction ... 360

